# Korean women’s perceptions of traumatic childbirth: a qualitative descriptive study

**DOI:** 10.1186/s12884-023-05986-8

**Published:** 2023-09-23

**Authors:** Jung Hee Yeo, Hae Sagong

**Affiliations:** 1 College of Nursing, Dong-A University, Busan, South Korea; 2 College of Nursing, Auburn University, 710 South Donahue Drive, Auburn, AL 36849 USA

**Keywords:** Trauma, Childbirth, Perception, Qualitative study, PTSD

## Abstract

**Background:**

Previous studies have established that negative or traumatic childbirth can create childbirth-related post-traumatic stress disorder (CB-PTSD). Because of the negative implications of CB-PTSD for mothers, children, and families, global qualitative research on traumatic or negative childbirth has risen in recent years. However, few studies have been conducted in South Korea. This study aims to explore women’s various perceptions of traumatic childbirth in South Korea.

**Methods:**

This qualitative descriptive study examined nine women who were at high risk of PTSD (IES-R-K > 24) at the time of the interview, between 1 and 11 years after childbirth. Semi-structured interviews were conducted. Interview transcripts were subjected to thematic analysis.

**Results:**

The analysis identified two themes with six subthemes, as follows: (1) person-centred factors (pain, guilt, maternal identity conflict, and damaged femininity); (2) society-centred factors (threatened dignity and disrupted relationships). These findings may be attributed to Korean culture (excessive motherhood and lookism), as well as unbearable pain, disrespectful childbirth environments, lack of spouse’s support, loss of their lifestyle, and unrealistic expectations.

**Conclusions:**

This study demonstrates various negative consequences, ranging from psychological damage to conflict in women’s relationships with their spouses, and others. This highlights the various perceptions stemming from traumatic childbirth and emphasizes the significance of clinical intervention. Therefore, healthcare professionals’ greater understanding of women’s perceptions and increased concern about childbirth and respectful childbirth environments are required. In addition, based on our findings, there is a need to develop interventions that can alleviate CB-PTSD and further improve women’s mental health, particularly through women-centred interventions.

## Background

Childbirth is traditionally viewed as a normal and positive experience, with evidence suggesting that women’s self-efficacy and self-esteem increase after childbirth [1]. However, according to previous studies, 34–54% of women perceive childbirth as traumatic [2, 3]. Additionally, 4.7% of women develop post-traumatic stress disorder (PTSD), and 12.3% develop post-traumatic stress symptoms following childbirth [4]. In Korea, a study reported that only 1.8% of postpartum women who gave birth to healthy babies were affected by PTSD [5], and there is currently no prior research targeting various cases.

There are various factors that could impact the development of childbirth-related (CB)-PTSD. A recent literature review reported that previous traumatic experiences, a lack of support, negative childbirth experiences, and post-partum depression were influencing factors of CB-PTSD [6]. Among these, traumatic or negative childbirth experiences primarily occur as a result of threats to the physical integrity, injury, and/or death of the mother and baby [7]. Such experiences can occur in various circumstances, including during normal labour [8], instrumental vaginal birth, and emergency caesarean birth [9]. As a result, the importance of these experiences is being emphasized. Moreover, the importance of childbirth experiences has increased further since CB-PTSD adversely affects the relationship between the mother and baby [10], delays post-partum adaptation [11], is highly comorbid to postpartum depression [12], and increases the abortion and caesarean birth rates in the next pregnancy [13]. Therefore, exploring the negative perceptions of childbirth may be the first step towards understanding and relieving CB-PTSD.

Global qualitative studies on traumatic or negative childbirth have risen in recent years, as shown by more studies focusing on specific traumatic events, such as perineal floor trauma and preterm labour [14, 15] and general traumatic childbirth [8, 13, 16]. Furthermore, there were studies of women who perceived their childbirth experience as traumatic even a few years prior [8, 17]. The former studies mainly identified situations or factors (i.e. vaginal examination, pain, low levels of support, dismissive attitude of clinical staff, medical culture, etc.) in which the traumatic childbirth experience occurred, and emotions and feelings at that time (i.e. fear, anxiety, feeling invisible, and out of control, etc.). The latter explored not only the results of the former, but also the impact of the traumatic childbirth experience on their personal life and relationships with their partner and child. These findings will constitute the basis for newly developed interventions for the prevention of CB-PTSD and new theories the long-term effects of traumatic childbirth experiences on women’s mental health.

However, since Korean society tends to regard childbirth as a woman’s social obligation and views it positively, studies focusing on addressing negative or traumatic childbirth are extremely rare. To date, there are only a few qualitative studies on women who have had multiple children or induced labour in South Korea [18, 19]. They have focused on both positive and negative aspects of childbirth and concluded that as precious and rewarding, although the negatives outweighed the positives. Therefore, the present study aimed to explore women’s perceptions of traumatic childbirth, to support healthcare professionals’ understanding of women with traumatic childbirth, and to develop strategies to improve women’s mental health in the future.

## Methods

### Design

A descriptive qualitative study design was used in this study to explore women’s perceptions of traumatic childbirth using semi-structured interviews and thematic analysis methods.

### Participants

This study included nine participants who lived in the cities of B or Y, South Korea. The inclusion criteria in the study were as follows: having never been hospitalized for complications of pregnancy or childbirth; having a single, healthy, and full-term baby. To collect rich data on traumatic childbirth, women with traumatic childbirth indicating a high risk of PTSD (i.e. a score > 24 out of 88 on the Korean version of Impact of Event Scale-Revised, IES-R-K [20]) were selected. Also, women undergoing uterine contractions, regardless of the mode of birth, were included in the study because the pain caused by uterine contractions may trigger traumatic childbirth. [8, 9, 17, 21]. The exclusion criteria encompassed individuals with pre-existing mental health issues, including pre-pregnancy depression or postpartum depression. This was determined by inquiring about their history of depression diagnosis or treatment, as well as whether they had personally experienced feelings of depression. In addition, individuals with cancer, chronic illnesses, or those who had experienced traumatic events such as domestic violence or sexual assault were excluded.

The nine participants’ characteristics were as follows: aged between 31 and 43 years (mean: 36, standard deviation: 4.75) all were college graduates; six of them had social occupations, including two nurses, one graduate student, one school teacher, one entrepreneur, and one private class teacher; all were married; five had a child; four had two children; and there were 13 births in total (vaginal, 8; caesarean, 5). The range of IES-R-K was 25–61 points. The ranges for the age of the first and second childbirth were 29–31 and 31–34 years, respectively. Additionally, considering South Korea’s cultural preference for sons, we included a questionnaire to assess participants’ satisfaction with their child’s gender. This was done to control for the potential influence of gender on traumatic experiences and all participants were satisfied with their child’s gender (Table 1). Time since the traumatic childbirth was less than 3 years for four participants, and between 4 and 11 years for five participants.

**Table 1:** Participants’ demographic and obstetric characteristics

Id	Age	Education	Employment	Marital status	Score of IES-R-K	Age at time of childbirth	Child’s gender	Mode of birth	Complications	Satisfaction with child’s gender
1	31	College	Yes	Married	52	29	Male	Caesarean birth after induced labour	-	Satisfactory
2	34	College	Yes	Married	57	30	Male	Vaginal birth	-	Satisfactory
						34	Female	Vaginal birth after induced labour	-	Satisfactory
3	35	Graduate	Yes	Married	46	31	Male	Caesarean birth after induced labour	-	Satisfactory
4	38	Graduate	Yes	Married	27	28	Female	Caesarean birth after induced labour	-	Satisfactory
5	42	College	Yes	Married	38	31	Female	Vaginal birth after induced labour	-	Satisfactory
						32	Male	Vaginal birth	Gestational diabetics	Satisfactory
6	34	College	No	Married	61	29	Male	Vaginal birth after induced labour	Mild perineal injury	Satisfactory
						31	Female	Vaginal birth after induced labour	-	Satisfactory
7	29	College	Yes	Married	37	27	Female	Vaginal birth	Mild perineal injury	Satisfactory
8	43	College	No	Married	25	28	Female	Caesarean birth after induced labour	-	Satisfactory
						32	Female	Vaginal birth	-	Satisfactory
9	39	College	No	Married	39	31	Female	Caesarean birth after induced labour	-	Satisfactory

IES-R-K: Korean version of Impact of Event Scale-Revised

### Procedure

Participants were recruited by a research assistant in compliance with the necessary ethical standards and received approval from the appropriate Institutional Review Board. All research methods employed in this study adhered to the relevant guidelines and regulations governing such investigations.

First, an advertisement to recruit participants was posted on a community website for mothers and at hospitals; purposive sampling was used to identify women who met the inclusion criteria. Potential participants were initially assessed for obstetric characteristics and the level of childbirth-related traumatic stress using the IES-R-K scale to determine their eligibility online a few days prior to the interview. Childbirth-related traumatic stress was evaluated using the IES-R-K [20], developed by Weiss and Marmar [22]. Information about this study and participants’ rights (right of withdrawal during the interview, clarification on data use purposes, methods of data disposal, and how participant anonymity would be preserved), consent, and questionnaires detailing general characteristics were undertaken through emails to online participants (Participants 6 and 7) or face-to-face interviews for offline participants (Participants 1, 2, 3, 4, 5, 8, and 9).

Semi-structured, face-to-face interviews were conducted for three months by the first author (who has conducted several qualitative studies on childbirth) using a pre-printed interview guide (15 November 2021–27 February 2022). The interview guide material was prepared through a review of previous studies [8, 16, 23] and it was reviewed by a qualitative research professor. For interviews, the main questions were as follows: Which aspects did you find traumatic?; what are your perceptions (emotions, thoughts, feelings, and physical perceptions) on traumatic childbirth during or/and after childbirth? During the interview, whenever the first author felt that there was a need for a deeper understanding of the issue, questions were asked to elaborate on the details. Participants were encouraged to talk freely and were reminded that they could discontinue answering if they found the externalisation of traumatic experiences distressing. Data collection continued until the participants’ experiences were repeated, corresponding to the state of data saturation. The data saturation level was determined by the first author and a qualitative research professor. Two participants were interviewed remotely at their comfortable time using live video (Zoom), and seven participants were interviewed face-to-face in one of the seminar rooms at the institution of the first author, ensuring a relaxed atmosphere without any interruptions. Interviews were conducted once for nine participants, while one participant was interviewed one more time to further confirm the data by phone. The duration of the interviews ranged from 50 min to 1.5 h. Interviews were recorded with the participant’s consent. Non-verbal representations, such as gestures, facial expressions, and body language, were recorded in field notes to accurately reflect the participants’ emotional state and capture vivid and authentic expressions. The first author repeatedly listened to the recorded interview data on the day of the interview and transcribed the participant’s data in coding notes.

### Data analysis

Thematic analysis was used to provide a purely qualitative account of the data that is richer and more detailed [24]. The thematic analysis was conducted as follows [25]: The first author repeatedly read the transcripts written in coding notes to familiarise herself with the dataset and then generated initial codes manually by examining the line-by-line transcripts. Initial codes are perception, emotion, thought, feeling-oriented words, or labels assigned to meaning units of text reflecting themes or topics that occur with regularity. Next, all codes were collated, and conceptually similar codes were organised into categories. This phase, which re-focuses the analysis on the broader level of themes rather than codes, involves sorting the different codes into potential themes and collating all the relevant coded data extracts within the identified themes [25]. These were then refined and reviewed by the other author and a qualitative research professor. They met periodically throughout the coding process to discuss and revise the coding scheme and potential themes. The themes were subsequently defined and further refined by a midwife and selected participants. This collaborative effort aimed to enhance the understanding and refinement of women’s childbirth perceptions. The midwife’s expertise, gained from their understanding of the childbirth environment and extensive observation of the childbirth process, contributed significantly to this process. The authors agreed with the confirmation of the theme. In the final step, two themes were formulated to describe women’s perceptions of traumatic childbirth.

### Rigor

This study secured trustworthiness based on credibility, dependability, transferability, and objectivity [25]. For credibility, the interviewer conducted several qualitative studies and was well-trained to interview. The authors checked with other experts (a midwife and qualitative research professor) to ensure that the findings were true. To ensure dependability, the interview was performed by one interviewer using a semi-structured interview guide, so data collection for all participants (i.e. by asking the same questions in the same order) was consistent. Triangulation analysis through the observations, interviews, and measures, was conducted. To ensure transferability, the authors describe the characteristics of the participants fully so that comparison with other groups could be made and potential threats to generalizability in sampling in the research limitation section could be described. To ensure objectivity, the authors described the study’s methods and procedures in explicit detail and shared the sequence of data collection, analysis, and presentation methods to create an audit trail.

## Results

Thematic analysis of interview transcripts generated two themes: (1) person-centred and (2) society-centred factors. The person-centred factor themes consisted of four subthemes of pain, guilt, maternal identity conflict, and damaged femininity. The society-centred factor themes consisted of two subthemes of threatened dignity and disrupted relationships. Table 2 shows the overview of the main themes and subthemes. Representative quotes for each subtheme are presented below.

**Table 2:** Overview of the main themes and subthemes

Theme	Subtheme
Person-centred factor	Pain
	Guilt
	Maternal identity conflict
	Damaged femininity
Society-centred factor	Threatened dignity
	Disrupted relationships

### Person-centred factors

The perceptions of participants focused on themselves. In other words, they expressed psychological damage related to unpleasant sensations (i.e. pain, vaginal examination, etc.), excessive motherhood, lack of support, and their shabby appearance.

#### Pain

The participants anticipated childbirth pain, but it exceeded their expectations. They attempted pain relief measures such as alternative painless childbirth methods, analgesics, and non-pharmacological methods. However, they expressed that the pain intensified progressively, eventually reaching a point where it felt uncontrollable:

‘After delivery, the pain suddenly became excruciating, as if my spine was being ripped apart. I was in so much pain that I couldn’t even make a sound. It felt like a car running over my abdomen. Painkillers were ineffective, and although I tried my best to endure it, it was unbearable.’ (Participant 4).

‘I screamed for someone to save me because the pain was unbearable. It felt like my flesh was tearing apart, and I felt terrible. I attempted to be strong to have a painless childbirth, but the pain persisted. At that moment, I thought that I might die and all I wanted was to escape from that situation.’ (Participant 5).

#### Guilt

The participants blamed themselves for not actually attempting to have a vaginal birth despite clear medical reasons for undergoing a caesarean section.

‘The third attempt at in vitro fertilization was successful, leading me to quit my job and seek out a hospital that offered Leboyer birth practices. So, I waited in expectation even with prenatal education, but the birth did not go well, so I eventually had surgery. Ah! I regretted that I did not have a vaginal birth, if only I had endured a little more at that time and wondered whether I had been a good mother to my child.’ (Participant 9).

‘I wanted to experience natural childbirth because my first child was delivered through surgery, without pain. So I had the thought of wanting to try it at least once [natural childbirth]. I was asking myself, wondering if I was being a coward or if I had too easily decided on the easier method for fear of pain. Did I do my best for the sake of my child?’ (Participant 1).

#### Maternal identity conflict

The participants endured excruciating pain while focusing solely on the baby’s well-being during childbirth, but they described losing their freedom as individuals due to childbirth, leaving only their role as a mother:

‘Though everybody celebrated after childbirth, I was feeling empty. Ah! What is worse is I am not the person I used to be. I’m done. I’m completely done. I don’t know who I am now. I have to take responsibility for this child and live bound by the child.’ (Participant 6).

‘Having a beautiful baby is one thing, but after childbirth, I can’t control my own time. [I used to] live happily before giving birth to a child. Childbirth is an experience that you don’t have to have. Do we have to do this hard work? I just want to make my dreams come true without any restrictions. After the child was born, I started to live someone else’s life, not my own.’ (Participant 4).

#### Damaged femininity

The participants felt embarrassed by their childbirth posture and exposed appearance during the childbirth process. After childbirth, they were disappointed by their shabby appearance, ultimately describing the disappearance of a women’s beauty and mystique:

‘I didn’t have a long dating period, so I didn’t want to show my naked body to my husband after giving birth, such as when I changed my pad or underwear…’ (Participant 3).

‘After childbirth, I wasn’t treated like a human. I had to wear a dress without my underpants; I had to open my shirt for breastfeeding, and my stool was on the pad; the childbirth posture was also weird…’ (Participant 7).

‘My face was swollen, my hair fell out, my pelvis is now larger, and my body shape has changed. I hated my reflection in the mirror.’ (Participant 2).

‘After childbirth, my body was ruined. I’m still overweight, with sagging skin, and I still have urinary incontinence. My appearance after childbirth has been stressful so far. Every time I have a chance, I still exercise like I’m obsessed. I liked to do make-up; I liked to wear heels, and I liked to dress up. But now, I don’t look like I did when doing these things as I did before, no matter how I do my make-up or dress up.’ (Participant 7).

### Society-centred factors

The perceptions of participants expanded from themselves to their relationships with society or other individuals. In other words, they reported damage in relation to their partners’ lack of support and the lack of respect from healthcare providers in the childbirth environment.

#### Threatened dignity

The participants anticipated giving birth in a pleasant childbirth environment and receiving special treatment from healthcare professionals and their partners. However, they perceived actual childbirth as being devoid of any psychological support in an environment that was no different from animals giving birth:

‘The healthcare professionals didn’t empathise with my pain, as they considered childbirth to be something familiar and routine for themselves. They treated all labouring women the same and disregarded them. It was disheartening when they coldly said, “If you can’t give birth with this level of pain, you’ll have to suffer more.” (omitted) I had multiple vaginal examinations during labour, and I didn’t want them. I wondered, “Why should I feel like there is a fist coming in during the vaginal examination? Why do they use such unhygienic and inhuman methods [for childbirth]? With the advancements in medicine, isn’t there any other way?”’ (Participant 5).

‘I felt like I wasn’t human. When the doctor was inserting their hand inside me and tearing me, it felt like I was being treated like an animal by a veterinarian. I was treated like a dog in labour. Childbirth is supposed to be noble and sacred, but I couldn’t find any trace of that in the delivery room.’ (Participant 8).

#### Disrupted relationships

The participants felt resentment towards their indifferent partners during the childbirth process; even after childbirth, their negative feelings towards their partners did not disappear, leading to ambiguity in their relationships.

‘When I look at other new mothers, I want to do everything for them. At first, my husband was somewhat responsive, but when I kept talking, he just thought it was painful. I want to empathise with everything the new mother says, I want to listen to her. The baby is really beautiful, but I didn’t want to see my husband at all. My husband did nothing during childbirth. I even thought about getting a divorce when I was recovering.’ (Participant 7).

‘Since my husband didn’t do anything during pregnancy and childbirth, and it’s all women’s work, he was just a stranger in that sense. We are becoming parenting comrades; we are not in love, and that makes me so sad.’ (Participant 6).

## Discussion

This study aimed to explore women’s perceptions of traumatic childbirth. This has from person-centeredness to their relationships with society. The person-centred factor themes included four subthemes of pain, guilt, maternal identity conflict, and damaged femininity; the society-centred factor themes included two subthemes of threatened dignity and disrupted relationships.

The ‘pain’ subtheme in this study was described in terms of fear, anxiety, and death, which were not controlled in any way. This finding aligns with a previous study demonstrating that women with higher traumatic stress symptoms expressed more pain and fear compared to their lower-trauma counterparts [26]. This was consistent with the findings of previous studies that explored the experiences of both women who gave birth preterm and had induced labour [18, 27] and those who had vaginal births [1, 13, 16]. Furthermore, a ‘lack of pain control’ or ‘unbearable pain’ was found to be a contributing factor to experiencing negative [16] or traumatic childbirth [8], which was supported in this study. In contrast, pain relief methods, such as deep breathing, walking, and distractions, were identified as important factors for women’s positive childbirth experiences [1]. Therefore, healthcare professionals should be aware of these experiences, acknowledge the significance of labour pain, and actively encourage and support women in pain management.

The ‘guilt’ subtheme refers to self-blame for the birth method that they chose (whether it had been vaginal birth or not). This finding aligns with the finding of Beck and Harrison’s [27] study on the experience of preterm births, where women felt solely responsible for the preterm birth. However, this experience was rarely identified in studies of full-term births [1, 13, 16]. In the present study of full-term births, this finding may be influenced by the concept of motherhood in Korean society. South Korea places significant emphasis on motherhood, emphasizing the protection and well-being of children [28]. The notion of ‘true motherhood’ is often perceived as enduring pregnancy and having a natural childbirth to ensure a healthy baby. In this study, women who did not follow this ideal doubted the authenticity of their motherhood or perceived themselves as inadequate mothers. These findings highlight the need to help women interpret motherhood in a feminist way that considers the identity, individuality, and independence of women, as well as changing social awareness of motherhood.

The ‘maternal identity conflict’ subtheme reflects dissatisfaction with life solely as a mother, rather than as an individual. In the present study, most participants struggled to fulfil their duty of giving birth, but experienced conflict regarding their maternal identity due to a loss of freedom, and ultimately questioned the worthiness of childbirth in a woman’s life. This finding is supported by previous studies that the traumatic childbirth experience is central to women’s identity and may threaten their perceptions of the self in their newly acquired maternal role [29]. Identity-related perceptions have also been mentioned in studies on primiparous mothers who have undergone traumatic childbirth [9]. While their result showed that women’s identities were challenged and altered because of their incompatibility with the maternal system, our finding may reflect the burden caused by childrearing and the thoughts of young Korean women who dream of having an economically-sound work life. Therefore, support for women’s childrearing seems to play a fundamental role in helping women establish their maternal identity and furthermore, may improve maternal-child attachments in the future.

The ‘damaged femininity’ subtheme refers to the loss of beauty and mystery symbolizing womanhood, as a result of body exposure during childbirth, weight gain, and the physical marks of pregnancy. This finding corroborated that of Kochan and Kabukcuoglu’s [14] qualitative study focusing on body image after childbirth for working women. Their finding indicated that women were disappointed and had a negative body image when their appearance and weight did not return to their pre-pregnancy state. Although this subtheme was not yielded in qualitative studies exploring traumatic childbirths [6, 8, 13, 27], it was significant in the present study. This finding shows how much Korean women value their appearance, and the discrepancy between the ‘real’ and ‘ideal’ regarding an individual’s appearance. Therefore, healthcare professionals should assist women in understanding the changes to their appearance and weight during and after pregnancy and provide comprehensive guidance and support for recovery programs to promote the restoration of femininity.

The ‘threatened dignity’ subtheme refers to not considering childbirth as a special event. This was similar to previous studies reporting that participants’ self-esteem was damaged due to the mistreatment that they received from healthcare providers, such as a lack of involvement in decision-making, dismissal of their concerns and preferences, and unprotected privacy [8, 9]. The subtheme in the present study may reflect the insincere attitudes of healthcare professionals and spouses, inhuman birth processes, and inadequate childbirth environments. The participants desired to be treated as special because they recognised childbirth as a profoundly rewarding event that contributes to families and society. They had expectations of physically and emotionally optimal childbirth environments. However, when the reality did not meet their expectations, their sense of dignity was compromised. Therefore, respectful childbirth environments are required to enhance women’s dignity, which will be addressed by improving the quality of maternity services and eliminating dehumanizing practices. Moreover, healthcare professionals should provide opportunities for women to become familiar with the childbirth environment.

The ‘disrupted relationships’ subtheme refers to strained relationships with spouses because of dissatisfaction with emotional support during childbirth. This finding was consistent with previous studies [6, 17] describing the disruption in women’s relationships with their spouses. One participant in the present study described that her spouse had avoided sex since she gave birth and showed a lack of sexual interest; this led to more difficulties in their relationship. This experience was in line with Ayers et al.’s [17] finding that loss of sexual intimacy contributed to a strain on relationships. To mitigate this experience, healthcare professionals need to provide women with opportunities for emotional communication with their spouses and participation in programs that aim to improve their marital relationships. Moreover, to prevent conflict in women’s relationships with their spouses, healthcare professionals should involve spouses in antenatal classes to learn supportive techniques and enhance their understanding of the challenges faced by mothers.

The overall findings of this study did not reveal any indication of the frequently observed sub-theme of a “negative relationship with the child” that had emerged from prior qualitative research on childbirth [17, 23]. This may be attributed to the culturally ingrained love that most Korean women have for their children, regardless of any traumatic childbirth experiences. Additionally, the qualitative descriptive approach employed in this study is characterized by lower levels of inference compared to other qualitative research methods such as phenomenology and grounded theory, allowing for a closer examination of participant data and maintaining a closer connection to the surface-level details of words and events [30]. Thus, this approach is considered an appropriate method for investigating perceptions of childbirth.

This study has several limitations that warrant highlighting. First, all participants were highly educated, had jobs during or before pregnancy or childbirth, and were married, so various differential general characteristics were not reflected in our sample. This is because participants were selected with more emphasis on conditions (high risk-PTSD, full-term birth, fertility history, satisfaction with child gender, etc.) other than these characteristics. Second, although participants had childbirth-related traumatic stress, they did not experience the main symptoms of PTSD, possibly because there were no severe traumatic memories or clinical CB-PTSD [29]. Another explanation may be related to memory and recall bias because of the timing of the interview, which was 1–11 years after childbirth.

## Conclusions

This study showed various negative consequences, from psychological damage to conflict in social relationships with spouses, colleagues, and children. These findings may be attributed to Korean culture (excessive motherhood and lookism), as well as unbearable pain, a disrespectful childbirth environment, the lack of spouse’s support, loss of their previous lifestyle, and unrealistic expectations. This highlights the various perceptions stemming from traumatic childbirth and the need for clinical intervention. Therefore, healthcare professionals’ greater understanding of women’s perceptions and their increased concern regarding childbirth and a respectful childbirth environment is required first. In addition, based on our findings, there will be a need to develop interventions that can alleviate CB-PTSD and further improve women’s mental health, particularly through women-centred interventions.

As previously mentioned, our findings were retrospective reports of childbirth experiences occurring 1 to 11 years ago, which may be influenced by the various psychological situations that the participants have experienced up to the interview time point. To restrict potential conflicts, the authors specifically focused on perceptions related to childbirth and made confirmations with participants to ensure that the quotes provided were indeed related to childbirth. However, prospective qualitative research is still needed to explore the impact of time after childbirth on women’s experiences of traumatic childbirth in the future. In addition, further quantitative research could compare psychological factors in women with and without CB-PTSD. This would provide the basis for supporting our findings and for developing specific interventions in clinical practice.

To our knowledge, this study was the first to explore traumatic childbirth perceptions among women in South Korea; thus, it may help develop interventions to prevent such negative experiences.

## Data Availability

The datasets supporting the conclusions of this article are included within the article.

## List of abbreviations

CB-PTSD: Childbirth-related post-traumatic stress disorder
IES-R-K: Korean version of Impact of Event Scale-Revised
